# Biotechnological production and application of the antibiotic pimaricin: biosynthesis and its regulation

**DOI:** 10.1007/s00253-015-7077-0

**Published:** 2015-10-29

**Authors:** Jesús F. Aparicio, Eva G. Barreales, Tamara D. Payero, Cláudia M. Vicente, Antonio de Pedro, Javier Santos-Aberturas

**Affiliations:** Area of Microbiology, Faculty of Biology, Universidad de León, 24071 León, Spain; Dynamique des Génomes et Adaptation Microbienne, UMR 1128, INRA, Université de Lorraine, 54506 Vandoeuvre-lès-Nancy, France; Department of Molecular Microbiology, John Innes Centre, Norwich Research Park, Norwich, NR4 7UH UK

**Keywords:** Antifungal agent, Cheese, Gene regulation, Keratitis, Metabolic engineering, Polyene macrolide, Preservative E-235, *Streptomyces*

## Abstract

Pimaricin (natamycin) is a small polyene macrolide antibiotic used worldwide. This efficient antimycotic and antiprotozoal agent, produced by several soil bacterial species of the genus *Streptomyces*, has found application in human therapy, in the food and beverage industries and as pesticide. It displays a broad spectrum of activity, targeting ergosterol but bearing a particular mode of action different to other polyene macrolides. The biosynthesis of this only antifungal agent with a GRAS status has been thoroughly studied, which has permitted the manipulation of producers to engineer the biosynthetic gene clusters in order to generate several analogues. Regulation of its production has been largely unveiled, constituting a model for other polyenes and setting the leads for optimizing the production of these valuable compounds. This review describes and discusses the molecular genetics, uses, mode of action, analogue generation, regulation and strategies for increasing pimaricin production yields.

## Introduction

Pimaricin (PIM), also called natamycin, tennecetin, natacyn and E235, is a natural product produced by given members of the genus *Streptomyces*, a class of filamentous soil-dwelling bacteria that undergo a complex life cycle involving differentiation and sporulation. It belongs to the polyene class of macrolide polyketides, and displays a strong and broad spectrum mould inhibition activity, yet being safe and effective at very low concentrations. Because its molecular target is ergosterol, an essential constituent of fungal membranes, and bacteria do not contain sterols in their membranes, it is inactive against bacteria. For the same reason, it is extremely reluctant to microbial resistance since the only way fungi would have to evade its action would be to change sterols from their membranes. For these reasons, and because of its low toxicity to mammalian cells, this molecule has become one of the major mould inhibitors used in the food industry. Its use was approved in 1967 as a cheese preservative, and since then, it has been extended to a wide variety of foods and beverages. In addition, PIM has been regarded as the most important agent in the management of fungal keratitis, a leading cause of blindness in corneal diseases, which is relatively common in warm climates and developing countries. Furthermore, it is also used as a crop protection agent to prevent mould contamination. Besides its antifungal action, it is also active against protozoa having ergosterol in their membranes.

Useful reviews about polyene macrolide biosynthesis and analogue generation are available in the literature (Aparicio et al. 2003; Aparicio et al. 2004; Caffrey et al. 2008; Kong et al. 2013). This mini-review provides a general view of the applicability of PIM, describes its particular mode of action and highlights the recent advances in the molecular genetics and metabolic engineering of its biosynthetic pathway to produce PIM derivatives with enhanced properties. It also provides important insights about the considerable progress attained in recent years aimed to unveil the complex network of regulators involved in its biosynthesis and discusses different strategies for increasing production yields.

### Discovery and structure

PIM is a small-sized polyene antibiotic. Its name derives from the South African region of Natal (‘Christmas’ in Portuguese, Vasco da Gama having landed on its shores on 25 December 1497), where the first producing strain, *Streptomyces natalensis*, was isolated (Struyk et al. 1957-1958). In addition, other strains like *S. gilvosporeus*, *S. lydicus* and *S. chatanoogensis* have also been identified as PIM producers.

**Fig. 1:**
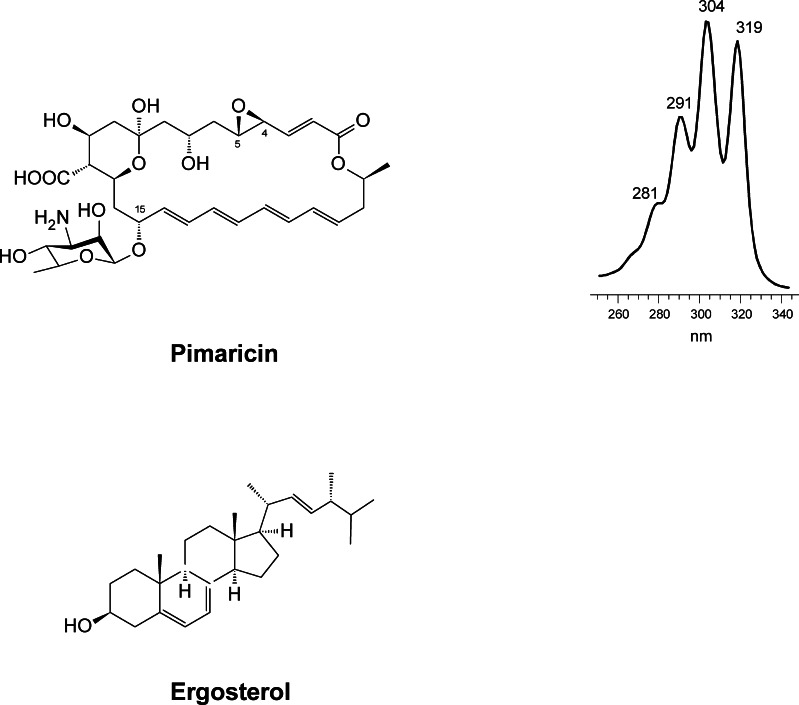
Structures of PIM and its target ergosterol. The UV-visible absorption spectrum of PIM is also included

Its structural core is a 26-membered macrolactone ring with four conjugated double bonds (chromophore) (Fig. 1). Its tetraene nature was unveiled shortly after its discovery (Patrick et al. 1958), but its correct covalent structure was not solved until 1966 (Golding et al. 1966) and its stereochemical structure some 24 years later (Lancelin and Beau 1990). More recently, its solution NMR structure has been described (Volpon and Lancelin 2002). Like most glycosylated polyenes, its molecule contains a mycosamine (3-amino-3,6-dideoxy-D-mannose) moiety linked to the macrolactone ring via a β-glycosidic bond at C15. In the aglycone, the most characteristic features are the presence of an epoxide group at C4-C5, which originates from a double bond (Mendes et al. 2001, 2005); an exocyclic carboxyl function at C12 that derives from a methyl group (Caffrey et al. 2008; Martín and Aparicio 2009; Qi et al. 2015; Liu et al. 2015b); and an internal hemiketal ring resulting from spontaneous cyclisation between a keto group at C9 and a hydroxyl group at C13 (Aparicio et al. 2000) (Fig. 1). Because of the presence of the chromophore in its structure, PIM shows characteristic physicochemical properties, including a strong UV-visible light absorption and photolability. The UV-visible light absorption spectrum of PIM shows a characteristic shape due to its multipeak pattern (Fig. 1). Like in all polyenes, the chromophore is located opposite to a number of hydroxyl functions, making PIM strongly amphipathic, the region where the chromophore lies has a planar and rigid lipophilic nature, whilst the hydroxylated region is typically flexible and hydrophilic (Bolard 1986). This feature allows the molecule to interact with the sterol molecules present in fungal cell membranes (predominantly ergosterol), by means of hydrophobic interactions between the hydrophobic portion of the polyene and the sterol, which results in cell death (Aparicio et al. 2004).

Because of its amphiphilic nature, it is poorly soluble in water and almost insoluble in non-polar solvents. As a powder, it is stable in the dark, with no loss of activity, but it is light sensitive in aqueous suspensions. Its CAS number is 7681-93-8.

### Bioactivity

PIM has a strong antifungal activity on most fungi (minimal inhibitory concentrations are in the micromolar range). For many years, it was thought that its mode of action on fungal membranes was to act as a permeabilising agent, as occurs with other polyenes (Bolard 1986). However, in clear contrast to large glycosylated polyenes like nystatin or amphotericin B that kill fungi by forming ion-permeable pores upon binding to ergosterol (Fujii et al. 1997; Baginski et al. 1997, 2006), or to the small polyene filipin which lacks the mycosamine moiety and also the carboxyl group, and is thought to interact with sterols by forming a sandwich-like structure embedded within the hydrophobic core of the lipid bilayer, which results in membrane fragmentation and cellular leakage (de Kruijff and Demel 1974; Knopik-Skrocka and Bielawski 2002), PIM blocks fungal growth by binding specifically to ergosterol but without permeabilising the membrane (te Welscher et al. 2008).

Thus, unlike other polyene antibiotics, PIM does not seem to form membrane disruption complexes and its action must be correlated with the alteration of the normal functioning of ergosterol in the fungal membrane. Ergosterol is the principal sterol in fungal cells, and has been correlated with multiple functions, including endocytosis, exocytosis, vacuole fusion, polarity and morphogenesis (Munn 2001; Kato and Wickner 2001; Heese-Peck et al. 2002; Martin and Konopka 2004; Wachtler and Balasubramanian 2006; Mysyakina and Funtikova 2007; Takeshita et al. 2008; Jin et al. 2008). Recently, Van Leeuwen et al. (2009) have proven that PIM inhibits endocytosis in germinating conidia of *Penicillium discolor* without causing extensive cell damage (i.e. without membrane permeabilisation), and Breukink and his group have also found that PIM impairs vacuole fusion via perturbation of ergosterol-dependent priming reactions that precede membrane fusion (te Welscher et al. 2010). Given that the priming phase consists solely on protein rearrangements, they pointed out to a more general mode of action such as the disturbance of ergosterol-dependent protein functions (te Welscher et al. 2010). This hypothesis was demonstrated only 2 years later, when they proved that PIM inhibits growth of yeasts and fungi via the immediate inhibition of amino acid and glucose transport across the plasma membrane by an ergosterol-dependent inhibition of transport proteins (te Welscher et al. 2012).

Because its molecular target is ergosterol, a structural constituent of fungal membranes, PIM, is extremely unlikely to provoke microbial resistance (Aparicio et al. 2004).

Besides its antifungal action, PIM has been involved in the immune response activation by triggering interleukin-1β secretion through activation of the NLRP3 inflammasome (Darisipudi et al. 2011). The mechanism of activation relies on the induction of potassium efflux from the cells as well as on phagocytosis-dependent lysosome destabilisation. This suggests that besides inhibiting fungal growth directly, it may also suppress fungal growth indirectly via activating innate host defence.

Additionally, this polyene has also been found to be active ‘in vitro’ against several protozoa such as *Trypanosoma* (Rolón et al. 2006) or *Acanthamoeba* (Sunada et al. 2014). These organisms have ergosterol-derived compounds as components of their membranes, making PIM or its derivatives also potentially useful as antiparasitic agents.

### Applications

#### Therapy

PIM has found clinical application as a topical agent in the treatment of various fungal infections, including oral, intestinal or vulvovaginal candidiasis (Cevher et al. 2008). But the most important playground for PIM is in the treatment of ophthalmic mycoses. PIM was the first antifungal agent approved by the Food and Drug Administration (FDA) of the United States (in 1978); it can be used for the treatment of fungal blepharitis, conjunctivitis, scleritis and endophthalmitis (Thomas 2003) and constitutes the first-line treatment in fungal keratitis (Ansari et al. 2013). It possesses activity against a great variety of yeast and filamentous fungal pathogens, including *Alternaria*, *Candida*, *Cephalosporium*, *Colletotrichum*, *Curvularia*, *Lasiodiplodia*, *Scedosporium*, *Trichophyton* and *Penicillium* spp. (Kaliamurthy et al. 2004; Thomas and Kaliamurthy 2013; Hsiao et al. 2014), and is currently considered the most effective medication against *Fusarium* and *Aspergillus* (Lalitha et al. 2007). Additionally, PIM is also effective for the treatment of keratitis produced by protozoa such as *Acanthamoeba* (Sunada et al. 2014).

#### Food

**Fig. 2:**
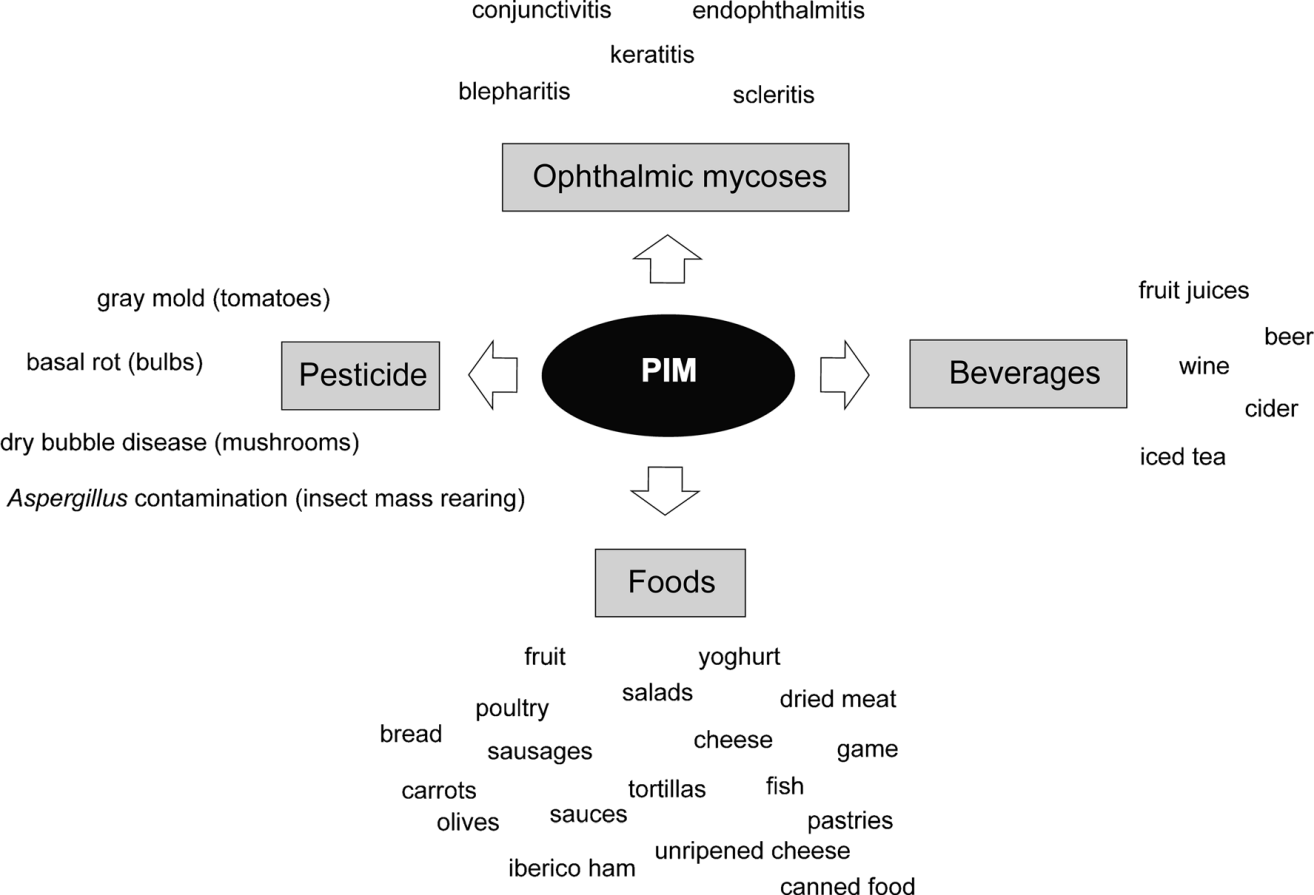
Applications of PIM

Because of its broad spectrum of activity, its low likelihood of causing microbial resistance and especially its low toxicity to mammalian cells (Arima et al. 2014), PIM has been widely used as a food preservative for more than 40 years (Fig. 2). It is significantly more effective than sorbate, another commonly used antifungal preservative (Shibata et al. 1991; Pipek et al. 2010). When applied on the surface of foods, it does not affect its organoleptic properties (taste, texture and colour), and has prolonged antimicrobial activity, being safe for consumption because its oral absorption is negligible (Juneja et al. 2012). It has been authorised by the European Food Safety Authority (EFSA) (additive E235), the World Health Organisation (WHO) and the FDA for protecting foods from yeast and mould contamination and possible inherent risks of mycotoxin poisoning. Notably, it is the only antifungal agent with a generally regarded as safe (GRAS) status.

Since bacterial membranes are devoid of sterols, PIM is not active against bacteria, thus making it an ideal antimicrobial during bacterial ripening and fermentation processes for fermented foods. Thus, it has been traditionally used as a preservative in cheese and cured sausage production (Stark 2003). It is used for the surface treatment of almost every type of cheese, either added as an emulsion for coating the cheese rind or applied by dipping or spraying. Under these conditions, PIM crystals remain on the surface of the product, and the soluble fraction hardly penetrates (Stark 2003), thus not interfering with the internal microorganisms that confer their organoleptic properties to these products. Sausages are treated with PIM by dipping or spraying to prevent fungal growth during ageing.

Besides its major application on cheese and sausages, PIM can also be used to prevent mould growth in yoghurt and other dairy products such as unripened cheese (e.g. cream cheese, cottage cheese and mozzarella) or whey protein cheese (e.g. ricotta), also in dried uncooked meats and in iberico and prosciutto hams. PIM can also be used to extend the shelf life of different fruit and vegetable preparations, salad mixes, baking products, sauces, fish, poultry, etc. (Fig. 2). Recently, PIM has been used to successfully inhibit the growth of fungi during natural black olive fermentation (Hondrodimou et al. 2011) and to prevent carrot spoilage in refrigerated storage facilities (de Vries et al. 2008).

Besides its direct application on foods, PIM has been successfully incorporated into different packaging films/coatings (de Oliveira et al. 2007; Pintado et al. 2010; Hanusová et al. 2010; Jiang et al. 2013b), including some that are edible (Fajardo et al. 2010), where it has proven to be gradually released over long periods of time, thus extending the shelf life of the product. This ‘active packaging’ is receiving great attention, since in several countries, there are restrictions on the direct use of PIM with certain foods.

#### Beverages

PIM has also been described to be very effective for controlling growth of *Aspergillus carbonarius*, the fungus responsible for contamination of wine, grapes and grape juice with ochratoxin A (one of the most abundant mycotoxins) (Medina et al. 2007). PIM is used to prevent fungal spoilage in other beverage products before their packaging, as it is effective at low concentrations, it is stable if kept protected from light and it is not affected by a wide range of pH values (Juneja et al. 2012). Regulations vary from one country to another but depending on the country; it may be used in fruit juices, beer, wine, cider or iced tea (Mann and Beuchat 2008; Siricururatana et al. 2013) (Fig. 2).

#### Pesticide

Furthermore, PIM is also used as a natural and safe product for crop protection. It is used to control various fungal diseases but especially basal rots on ornamental bulbs such as daffodils that are caused by *Fusarium oxysporum* (Copping and Duke 2007). Its efficacy to control tomato gray mould disease caused by *Botrytis cinerea* in greenhouse conditions has also been reported (Lu et al. 2008). In 2012, it has been approved as a biopesticide by the US Environmental Protection Agency for its use in enclosed mushroom production facilities to prevent dry bubble disease caused by *Lecanicillium fungicola*, a devastating pathogen in the mushroom industry. Recently, its efficacy controlling *Aspergillus niger* contamination in rice leaffolder larvae (*Cnaphalocrocis medinalis*, a *Lepidoptera* important for rice growth) mass-rearing facilities has been reported (Su et al. 2014).

Other applications include its use in selective growth media to prevent growth of yeast and filamentous fungi, such as in the isolation of *Brucella* (Stack et al. 2002) or *Legionella* spp. (Edelstein and Edelstein 1996).

### Biosynthesis and export

PIM, like other macrocyclic polyketides, is synthesised by the action of so-called type I modular polyketide synthases (PKSs) (Aparicio et al. 2003) that sequentially assemble carbon chains from small acyl precursors, in a fashion that mechanistically resembles fatty acid biosynthesis. But, whereas in fatty acid biosynthesis, each elongation step is followed by a complete set of reactions including ketoreduction, dehydration and enoylreduction; in the synthesis of PIM and other macrolides, the product of each decarboxylating condensation may undergo all, some or none of the abovementioned modifications, thus resulting in alkanes, double bonds, hydroxyl groups or ketones at defined positions of the polyketide chain. Finally, a thioesterase domain located at the C-terminal end of the last PKS (PimS4) releases the chain by lactonisation. Once the first macrolide intermediate is formed (pimaricinolide), it becomes the substrate of three functionalisation enzymes that carry out specific oxidations and a glycosylation to yield the final PIM molecule (see below).

Biosynthetic genes from *S. natalensis* were the first described for a polyene macrolide (Aparicio et al. 1999), and since then, the whole cluster responsible for the biosynthesis of PIM (*pim*) from 12 acetate and one propionate units has been thoroughly studied (Martín and Aparicio 2009) (Table 1). The cluster from another PIM producer such as *S. chattanoogensis* has been described later and named *scn* (Du et al. 2011a). The cluster from *S. natalensis* is divided into two subclusters which encode a highly complex PKS distributed in five multifunctional polypeptides (PimS0 to PimS4) harbouring 13 homologous sets of enzyme activities (modules) and a total of 60 catalytic domains (Aparicio et al. 2000). The DNA region contains 15 additional open reading frames which govern post-PKS modification of the polyketide skeleton, export and regulation of gene expression (Table 1).

**Table 1:**
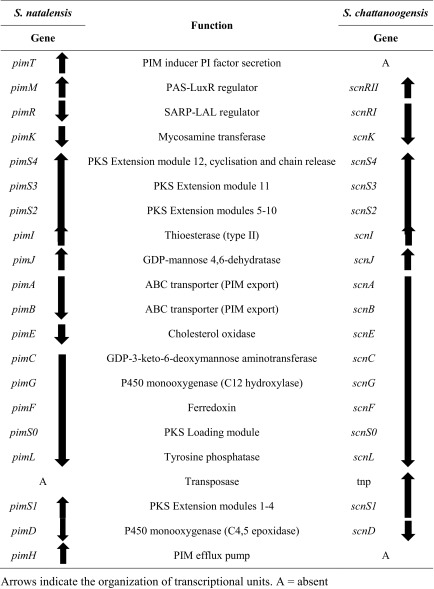
Comparison of PIM clusters and transcriptional units from *S. natalensis* and *S. chattanoogensis*

Both clusters show a large degree of synteny. Compared with the *scn* cluster, the *pim* cluster contains only two major strain-specific differences, which correspond to *pimH*, an efflux pump-encoding gene (Aparicio et al. 2000), and *pimT* that encodes an amino acid exporter (Vicente et al. 2009) involved in modulating PIM production via secretion of the PIM inducer 2,3-diamino-2,3-bis(hydroxymethyl)-1,4-butanediol (PI factor) (Recio et al. 2004). Both genes are located at the two ends of the cluster (Table 1). In turn, the *scn* cluster contains a putative transposase gene located downstream from *scnS1*, the PKS in charge of the first elongation step in polyketide construction. Interestingly, although the coding regions of the two clusters are highly conserved, the intergenic regions are much more variable, thus accounting for a different transcriptional organisation between them (Du et al. 2011a) (Table 1).

**Fig. 3:**
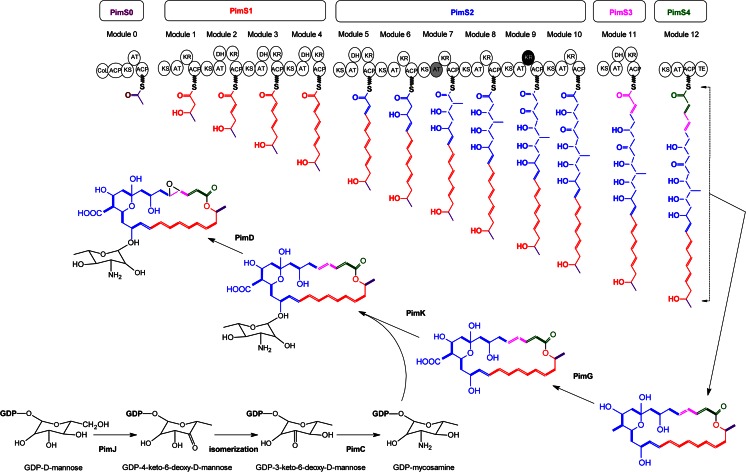
Biosynthesis of PIM. *Each circle* represents an enzymatic domain. *ACP* acyl carrier protein, *AT* acyltransferase, *CoL* carboxylic acid:CoA ligase, *DH* ß-hydroxyacyl-thioester dehydratase, *KR* ß-ketoacyl-ACP reductase, *KS* ß-ketoacyl-ACP synthase, and *TE* thioesterase. The KR domain in *black* (module 9) is predicted to be inactive. The AT in module 7 (*grey*) is predicted to incorporate a propionate extender unit. Biosynthetic pathway for mycosamine is also included. The isomerisation step is thought to be spontaneous

The construction of PIM macrolide skeleton is initiated by PimS0 and ends at PimS4 that thanks to its terminal thioesterase domain releases the growing chain from the enzyme and cyclises it to yield a 26-membered macrocyclic lactone (pimaricinolide) (Fig. 3). The fidelity of chain construction has been demonstrated to be dependent on *scnI* (the *pimI* orthologue from *S. chattanoogensis*). This discrete thioesterase plays an important editing role in the selection of starter acyl units and in the removal of aberrant extender units during PIM biosynthesis (Wang et al. 2014a). It can be complemented by other discrete thioesterases (Wang et al. 2014a), and it is conceivable that it could also have a complementary role with the terminal thioesterase domain of PimS4. Once the aglycone is synthesised, a cytochrome P450 enzyme, *pimG*, was proposed to catalyse an oxidation of the exocyclic methyl group at C12 via triple hydroxylations, to form a carboxyl group, yielding 12-carboxypimaricinolide (Martín and Aparicio 2009). This hypothesis has recently been proven by inactivation of *pimG* orthologue *scnG* form *S. chattanoogensis* L10 (Liu et al. 2015b). Then, the amino sugar mycosamine is attached to the macrocyclic aglycone at C15 by the glycosyltransferase *pimK* to yield 4,5-deepoxypimaricin (Fig. 3) (Mendes et al. 2005; Liu et al. 2015b), and finally, another P450 monooxygenase, *pimD*, catalyses the last oxidation step to yield an epoxy group between C4 and C5 from a double bond generated by the PKS (Mendes et al. 2001, 2005). The X-ray structure of this latter P450 has been resolved, both substrate-free and in complex with 4,5-deepoxypimaricin (Kells et al. 2010).

The mycosamine sugar is synthesised from guanosine diphosphate (GDP)-mannose derived from fructose-6-phosphate (Nic Lochlainn and Caffrey 2009). Inactivation of *pimJ* orthologue *amphDIII* in the amphotericin-producing strain *S. nodosus* resulted in accumulation of amphoteronolides demonstrating that mycosamine is biosynthesised from GDP-mannose (Byrne et al. 2003), and heterologous expression of the orthologue from nystatin biosynthesis *nysDIII* has proven that the first step in the mycosamine-specific pathway is carried out by GDP-mannose 4,6-dehydratase (Nedal et al. 2007) (Fig. 3). Thus, *pimJ* is proposed to catalyse conversion of GDP-mannose to GDP-4-keto-6-deoxymannose, which presumably undergoes a spontaneous 3,4-isomerisation to give GDP-3-keto-6-deoxymannose (Aparicio et al. 2003), the substrate of the aminotransferase *pimC* for biosynthesis of GDP-3-amino-3,6-dideoxymannose (GDP-mycosamine). Finally, the glycosyltransferase *pimK* catalyses the attachment of mycosamine to the pimaricinolide (Mendes et al. 2005).

Due to its mode of action, PIM does not have any antibacterial activity and thus should not represent a threat to the producing organism; however, its accumulation inside the cell may be harmful, which suggests that producing organisms should ensure an active efflux. The products of three genes in the cluster might be involved in PIM export in *S. natalensis*. Two of them belong to type III ATP-binding cassette (ABC) transporter proteins, which combine both ATPase and transmembrane domains within the same polypeptide (Mendez and Salas 2001). These, *pimA* and *pimB*, are thought to associate forming a heterodimer (Aparicio et al. 2003), and their role in PIM secretion has been deduced upon inactivation of the *pimA* and *pimB* homologues, *nysH* and *nysG*, which encode the nystatin ABC heterodimer transporter. Such mutants still exported some amount of nystatin, suggesting the existence of alternative transport systems for this antifungal antibiotic (Sletta et al. 2005). Such alternative transport system, in the case of PIM, could be constituted by the putative efflux pump encoded by *pimH* (Aparicio et al. 2000).

### Metabolic engineering and semi-synthetic derivatives

In the search for improvement of PIM properties, a good number of semi-synthetic derivatives have been described in the literature, including esters (Bonner et al. 1972; Falkowski et al. 1979), N,N-dialkyl (Paquet and Carreira 2006), N-alkyl (Suloff et al. 2003), N-acyl (Nirgudkar et al. 1988), N-glycosil (Falkowski et al. 1980), N-aryl (Belakhov et al. 2010) and hydrophosphoryl derivatives (Belakhov et al. 2008), amongst others. Liposomal preparations have been also studied (Bouaoud et al. 2015).

Moreover, the knowledge of PIM biosynthetic pathway, and the development of an efficient conjugation system for *S. natalensis* (Enríquez et al. 2006), has allowed the generation, by genetic manipulation, of a number of less toxic or more water-soluble derivatives (Caffrey et al. 2008; Kong et al. 2013).

**Fig. 4:**
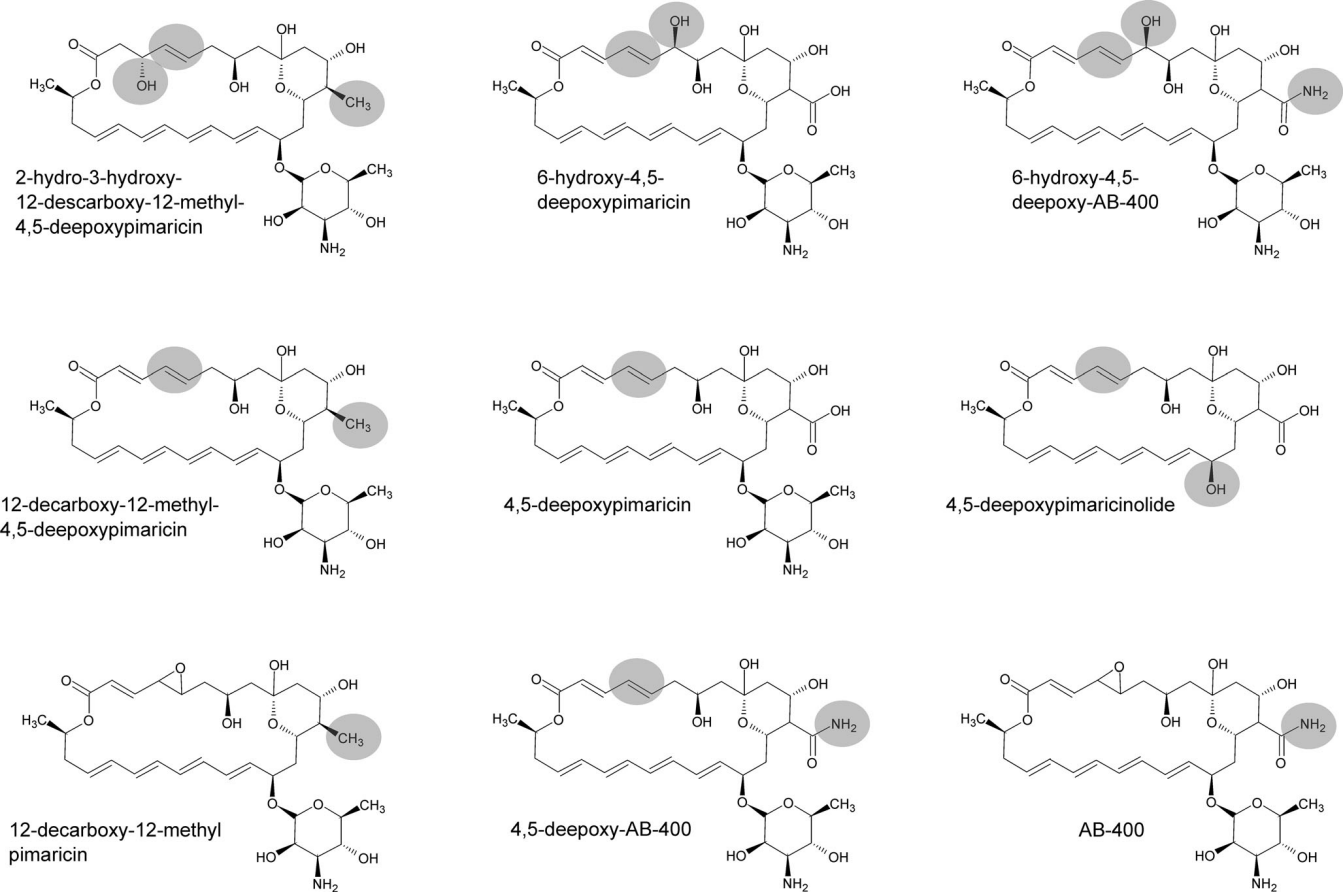
PIM derivatives obtained by metabolic engineering of the biosynthetic gene cluster. The *grey circles* highlight structural differences from the original PIM molecule

The first PIM derivative obtained by genetic engineering of *S. natalensis* was its immediate precursor, 4,5-deepoxypimaricin (DEP) (Mendes et al. 2001). This was obtained by disruption of *pimD*, the gene encoding the cytochrome P450 monooxygenase responsible for epoxidation at C4-C5 that constitutes the last step of PIM biosynthesis (Mendes et al. 2005; Kells et al. 2010). This compound showed a clear decrease in antifungal activity, thus highlighting the importance of the epoxide group in the interaction of PIM with the fungal membrane. Recently, it has been shown that DEP can be accepted as a substrate by *nysL*, the *pimD* homologue responsible for the introduction of the last hydroxylation in the biosynthesis of nystatin (Volokhan et al. 2006), thus generating 6-hydroxy-4,5-deepoxypimaricin (6-OH-DEP) (Santos-Aberturas et al. 2015), which does not show an improvement in antifungal activity compared to DEP. Additionally, it has been shown that both DEP and 6-OH-DEP can undergo carboxamidation reactions catalysed by the amidotransferase PscA, thus resulting in carboxamidated derivatives (4,5-deepoxy-AB-400 and 6-hydroxy-4,5-deepoxy-AB-400, respectively; see Fig. 4) exhibiting improved antifungal activity in comparison with their carboxylated precursors (Santos-Aberturas et al. 2015). PscA and its homologous protein, PscB, are responsible for the natural occurrence of tetraene carboxamidated compounds (Seco et al. 2005, 2010; Miranzo et al. 2010), including AB-400, the PIM carboxamidated derivative (Cañedo et al. 2000). PscA has shown a much broader substrate that scope PscB, a fact that makes it a stronger candidate for the development of future polyene derivatives (Seco et al. 2010; Santos-Aberturas et al. 2015).

The recent manipulation of the PIM biosynthetic pathway in *S. chattanoogensis* has led to the production of new PIM derivatives by knocking out *scnG*, which encodes the P450 monooxygenase (homologous to *pimG*) proposed to be responsible for the formation of the carboxyl exocyclic group (Martín and Aparicio 2009) (Fig. 3). This gene disruption resulted in the accumulation of 4,5-deepoxy-12-decarboxy-12-methyl PIM, thus indicating that glycosyltransferase *scnK* is able to add the mycosamine moiety to the decarboxylated aglycone whilst *scnD* is unable to introduce the epoxide group (Fig. 3) (Liu et al. 2015b). In contrast, the inactivation of the same gene by Qi et al. (2015) resulted in accumulation of 12-decarboxy-12-methyl PIM in addition to that of 4,5-deepoxy-12-decarboxy-12-methyl PIM (Fig. 4), thus suggesting that *scnD* might be able to introduce the epoxide group to some extent. 12-Decarboxy-12-methyl PIM might be interesting since it shows decreased haemolytic effects together with a twofold increase of the original PIM antifungal activity levels (Qi et al. 2015). These authors have also characterised the bioactivity of 4,5-deepoxy-12-decarboxy-12-methyl PIM, showing that it maintains the PIM antifungal activity whilst exhibiting a decreased haemolytic effect. Interestingly, the mutant obtained by Qi et al. (2015) also accumulated the non-antifungal and non-haemolytic 2-hydro-3-hydroxy-4,5-deepoxy-12-decarboxy-12-methyl PIM (Fig. 4), suggesting that the *scnG*-catalysed carboxylation might occur during the PKS assembly and not after it. In addition, the disruption of *scnK* in *S. chattanoogensis* (Liu et al. 2015a, b) led to the accumulation of the 4,5-deepoxypimaricinolide aglycone (Fig. 4), which shows no antifungal activity by itself but could constitute an interesting scaffold for the introduction of alternative sugar moieties by semi-synthetic, mutasynthetic or protein engineering approaches. The accumulation of that intermediate supports the idea that *scnG* (*pimG*) acts before the glycosylation step in the PIM biosynthetic pathway (Fig. 3).

### Regulation

As occurs with most secondary metabolites synthesised by *Streptomyces*, PIM production takes place in a growth-dependent manner and is governed by complex regulatory networks that respond to population density and a variety of environmental and physiological signals (van Wezel and McDowall 2011; Liu et al. 2013). One of the key players of these networks is transcriptional regulation.

#### Transcriptional control by cluster-situated regulators

Transcriptional regulation is a complex process involving multiple signals and an intricate network of regulators that cross talk with each other. Typically, the lowest level is played by so-called pathway-specific transcriptional regulators, which are encoded within the respective biosynthetic gene clusters. Two transcriptional regulators are encoded by the PIM gene cluster, *pimR* and *pimM*.

*PimR* was the first pathway-specific transcriptional regulator of PIM biosynthesis to be described and also the first of its class (Antón et al. 2004). It is a transcriptional activator (knockout mutants fail to produce PIM) with a peculiar architecture. It combines an N-terminal streptomyces antibiotic regulatory protein (SARP) DNA-binding domain with a C-terminal half homologous to guanylate cyclases and large ATP-binding regulators of the LuxR family (LAL) (Antón et al. 2004). The C-terminal half includes the ATP/GTP-binding domain characteristic of these protein families but lacks the characteristic signature sequence at the N-terminus of guanylate cyclases or the LuxR-type helix-turn-helix (HTH) motif for DNA-binding present at the C-terminus of LAL regulators (Guerra et al. 2012). Recently, we have characterised *pimR* mode of action by means of electrophoretic mobility shift assays (EMSAs), DNaseI protection studies, reverse transcription quantitative PCR (RT-qPCR) and gene promoter replacement experiments and determined that it binds a single operator that contains three heptameric direct repeats of the consensus CGGCAAG with 4-bp spacers (Santos-Aberturas et al. 2012). Such operator lies in the promoter region of *pimM*, whose expression is activated upon *pimR* binding. Interestingly, the binding sequence of *pimR* (TGGCAAGAAAGCGGCAGGTGTTCGGCAAG) is exactly conserved in the intergenic region between *scnRII* and *scnRI* in the *scn* gene cluster of *S. chattanoogensis* (*pimM* and *pimR* counterparts, respectively (Du et al. 2011a)) and also between *pteF* and *pteR*, the corresponding counterparts in the filipin gene cluster of *S. avermitilis*, including the inter-heptamer nucleotides.

Regulators with a similar architecture include the orthologues *pteR* and *filR* involved in filipin biosynthesis in *S. avermitilis* (Ikeda et al. 2003) and *S. filipinensis*, respectively (Payero et al. 2015), but also regulators of peptidyl nucleoside antibiotic biosynthesis such as nikkomycins (*sanG*) and polyoxins (*polR*) (Liu et al. 2005; Li et al. 2009). Filipin is a pentaene macrolide, whilst nikkomycins and polyoxins are peptidyl nucleoside antibiotics. Interestingly, all these compounds are effective antifungals, and it has been speculated that the domain arrangement of these regulatory proteins might be related with the detection of common signals involved in the triggering of antifungal production (Santos-Aberturas et al. 2012). Noteworthy, the consensus heptamer for *pimR* is identical to those of *sanG* (He et al. 2010) and *polR* (Li et al. 2009), although in these cases, only two heptameric repeats are present in the operator.

**Fig. 5:**
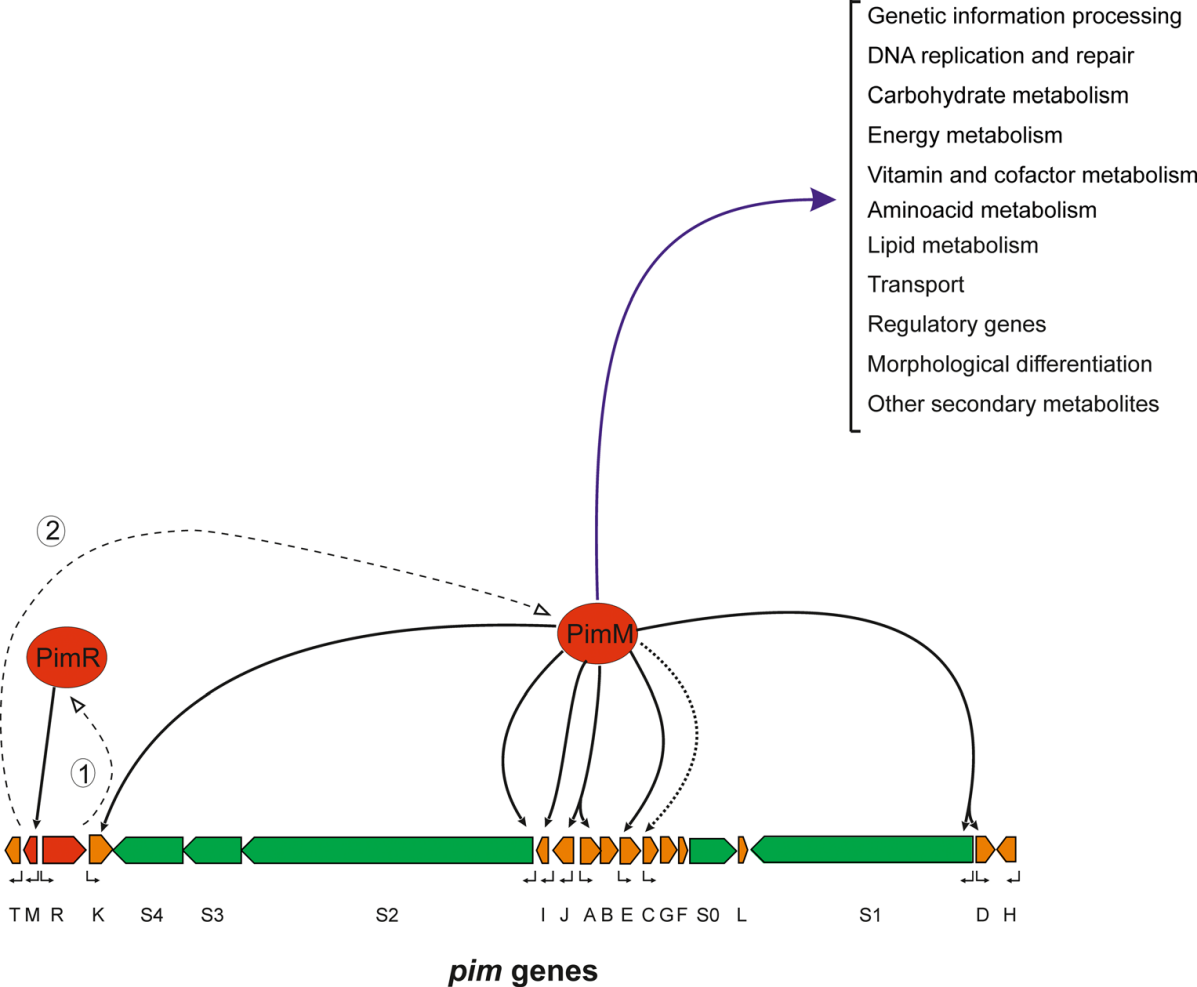
Model of PIM regulation. Proposed model for gene expression activation by the cluster-situated regulators *pimR* and *pimM*. The *pointed boxes* indicate the direction of transcription of *pim* genes. The transcriptional regulators are indicated in *red*, the PKS genes are shown in *green* and the remaining genes in *orange. Dashed lines* represent transcription and translation of regulatory genes. *Numbers* indicate the order of events. *Continuous black arrows* indicate direct transcriptional activation, whereas the *dotted arrow* indicates indirect activation. *Bent arrows* below the genes indicate transcriptional units. The *blue line* indicates other processes directly affected by *pimM* (Vicente et al. 2015)

*PimM* was the second transcriptional activator of PIM biosynthesis to be described (PIM production is abolished in deleted mutants) (Antón *et al*. 2007). It also has a peculiar architecture, combining an N-terminal PAS sensory domain (Hefti et al. 2004) with a C-terminal helix-turn-helix motif of the LuxR type for DNA binding (Santos et al. 2012). The PAS domain detects a physical or chemical stimulus and regulates, in response, the activity of the effector domain (Möglich et al. 2009). Unlike most other sensors, proteins containing PAS domains are located in the cytosol, and therefore, they detect internal signals, but they can also sense environmental factors that cross the cell membrane. In contrast with the majority of prokaryotic PAS domain-containing regulators, which are sensor kinases of two-component systems (Taylor and Zhulin 1999), *pimM* does not belong to a two-component system. Recently, we have characterised the mode of action of *pimM* at the molecular level, and determined the canonical binding site of this regulator as CTVGGGAWWTCCCBAG, just at the −35 hexamer of regulated promoters in *S. natalensis* (Santos-Aberturas et al. 2011b). The *pimM* paradigm is particularly attractive because homologous regulatory proteins have been found to be encoded in all known biosynthetic gene clusters of antifungal polyenes, and they have been shown to be functionally equivalent, to the extent that the production of PIM is restored in *S. natalensis* Δ*pimM* upon introduction of heterologous regulators of the PAS-LuxR class, such as *amphRIV* (amphotericin), *nysRIV* (nystatin) or *pteF* (filipin) into the strain (Santos-Aberturas et al. 2011b). Furthermore, introduction of a single copy of *pimM* into the amphotericin-producing strain *S. nodosus*, into the filipin-producing strain *S. avermitilis* or into the rimocidin-producing strain *S. rimosus* boosted the production of all polyenes, thus indicating that these regulators are fully exchangeable (Santos-Aberturas et al. 2011b). *PimR* and *pimM* act in a hierarchical way, *pimR* binds *pimM* promoter and activates its transcription (Santos-Aberturas et al. 2012) and the gene product of the latter activates transcription from eight different promoters of PIM structural genes directly (Santos-Aberturas et al. 2011a) (Fig. 5).

Although *pimM* has been traditionally considered as a PIM-specific regulator, recent results have shown that it cannot be considered as pathway-specific but as a regulator with a wider range of implications. Its canonical operator was used to search for putative targets of orthologous protein *pteF* in the genome of *S. avermitilis*, finding 101 putative operators, 97 outside the pentaene filipin gene cluster (*pte*). These binding sites were located inside or upstream from genes involved in different aspects of both primary and secondary metabolism, including genetic information processing, DNA replication and repair, energy metabolism, carbohydrate metabolism, lipid metabolism, morphological differentiation, transcriptional regulation and secondary metabolite biosynthesis, amongst others (Fig. 5), thus suggesting that the regulator could govern those processes. Seventeen of these operators were selected, and their binding to *pimM* DNA-binding domain was demonstrated by EMSA (Vicente et al. 2015). As a proof of concept, the biosynthesis of the ATP-synthase inhibitor oligomycin whose gene cluster included two operators was studied. *PteF* mutants, which show a severe loss of filipin production and delayed spore formation in comparison to that of the wild-type strain (Vicente et al. 2014), also showed a severe loss of oligomycin production and reduced expression of *olm* genes, whereas gene complementation of the mutant restored phenotype, thus demonstrating that *pteF* was able to co-regulate the biosynthesis of two related secondary metabolites, filipin and oligomycin. This cross regulation could therefore be extended to all the processes indicated above, which suggests that PAS-LuxR regulators affect a plethora of processes previously unforeseen.

#### Global regulatory mechanisms

The biosynthesis of PIM in *S. natalensis* is very sensitive to repression by inorganic phosphate. Concentrations as low as 2 mM are sufficient to block PIM production. This negative effect is exerted at the transcription level (Mendes et al. 2007b). The cellular response to phosphate scarcity is driven by the two-component system *phoR*-*phoP. PhoR* is a membrane-bound sensor kinase, and *phoP* is a DNA-binding response regulator that controls transcription of target genes known as the *pho* regulon. DNA binding of phosphorylated *phoP* to its operators (PHO boxes) takes place following phosphate depletion. The *phoU*-*phoR-phoP* region of *S. natalensis* has been characterised (Mendes et al. 2007b). The *phoP* protein from *S. coelicolor* binds to PHO box consensus sequences in the *S. natalensis phoU-phoRP* intergenic region, indicating that the system is autoregulated. Several *pim* genes showed increased expression in the *phoP*-disrupted mutant, but no consensus PHO boxes were found in the whole *pim* cluster, suggesting that phosphate control of these genes is mediated by *phoP* via other regulators.

Another pleiotropic regulator which positively regulates PIM production by direct binding to the promoters of structural genes has been described in *S. chattanoogensis. WhiG* binds to the promoters of *scnC* and *scnD* (homologues to *pimC* and *pimD*, respectively) (Liu et al. 2015a). This regulator also acts as an activator of *scnRI* and *scnRII* transcription (orthologues of *pimR* and *pimM*, respectively), although in this case indirectly (Liu et al. 2015a).

Other wide domain regulators that have been involved in indirect PIM regulation are the *adpA* pleiotropic regulator, which has been shown to act as a positive regulator of PIM production in *S. chattanoogensis* (Du et al. 2011a), and one of its direct targets *wblA* (Yu et al. 2014).

PIM production is an aerobic process and thus positively affected by oxygen availability. However, high levels of molecular oxygen consumption can lead to the formation of reactive oxygen species (ROS) that can damage cell components. The redox-based regulation network triggered by an imbalance of the intracellular ROS homeostasis, in particular intracellular H_2_O_2_ levels, is also able to modulate the biosynthesis of PIM in *S. natalensis* (Beites et al. 2011). Inactivation of superoxide dismutase *sodF* leads to reduced PIM production, whilst suppression of the H_2_O_2_-detoxifying enzymes like the alkyl hydroperoxidase system *ahpCD* or the catalase *katA1* provokes a PIM overproducer phenotype (Table 2), thus suggesting a positive correlation between intracellular H_2_O_2_ and PIM production (Beites et al. 2011). Recently, a cross talk between phosphate metabolism and oxidative stress in *S. natalensis* has been unveiled by transcriptome analysis. Deletion of either *sodF* or *ahpCD* causes a delay on the transcriptional activation of the *pim* cluster, most likely attributable to a delay on Pi exhaustion in the culture broth. Additionally, these authors have identified cellular NADPH/NADH ratio and the availability of biosynthetic precursors via the branched chain amino acid metabolism as the main PIM biosynthetic bottlenecks under oxidative stress conditions (Beites et al. 2014).

**Table 2:** PIM production improvement strategies and yield

Strategy	Strain	Yield (%)	Reference
*PimM* overexpression (NP)	*S. natalensis*	240	Antón et al. 2007
*ScnRII* overexpression (NP)	*S. chattanoogensis*	460	Du et al. 2009
*SlnM* overexpression (NP)	*S. lydicus*	190	Wu et al. 2014
*SlnM* overexpression (ermE*P)	*S. lydicus*	240	Wu et al. 2014
*SlnM* overexpression (NP + ermE*P)	*S. lydicus*	300	Wu et al. 2014
*SngA* overexpression (NP)	*S. natalensis*	170	Lee et al. 2008
*WblA* overexpression (ermE*P)	*S. chattanoogensis*	130	Yu et al. 2014
*WhiG* overexpression (ermE*P)	*S. chattanoogensis*	126	Liu et al. 2015a
*SchPPT* overexpression (ermE*P)	*S. chattanoogensis*	140	Jiang et al. 2013a, b
*SngR* deletion	*S. natalensis*	460	Lee et al. 2005
*PhoRP* deletion	*S. natalensis*	180	Mendes et al. 2007b
*AhpCD* deletion	*S. natalensis*	130	Beites et al. 2011
*KatA1* deletion	*S. natalensis*	156	Beites et al. 2011
Glycerol addition (100 mM)	*S. natalensis*	250	Recio et al. 2006
Pi factor addition (300 nM)	*S. natalensis*	133	Recio et al. 2004
Acetate:propionate addition (7:1) (2 g/L)	*S. natalensis*	250	Elsayed et al. 2013
Propanol addition (0.2 %)	*S. natalensis*	117	Li et al. 2014
Genome shuffling	*S. gilvosporeus*	197	Luo et al. 2012
Integration of *vgb* gene	*S. gilvosporeus*	407	Wang et al. 2014b
*Aspergillus niger* extracellular extract	*S. natalensis*	250	Wang et al. 2013
*Penicillium chrysogenum* extracellular extract	*S. natalensis*	300	Wang et al. 2013

The maximum yield is indicated

*NP* native promoter

#### Quorum-sensing signals

Quorum sensing is a communication mechanism that allows bacteria to detect a high density of population and react by different mechanisms of adaptation. Growing cells produce extracellular signals (often called autoinducers) that are detected by the remaining cells of the culture, which in turn respond to this stimulus by the transcription or repression of given genes. In *Streptomyces* sp., both morphological differentiation and secondary metabolite biosynthesis are controlled by quorum-sensing signals that act at nanomolar concentrations.

**Fig. 6:**
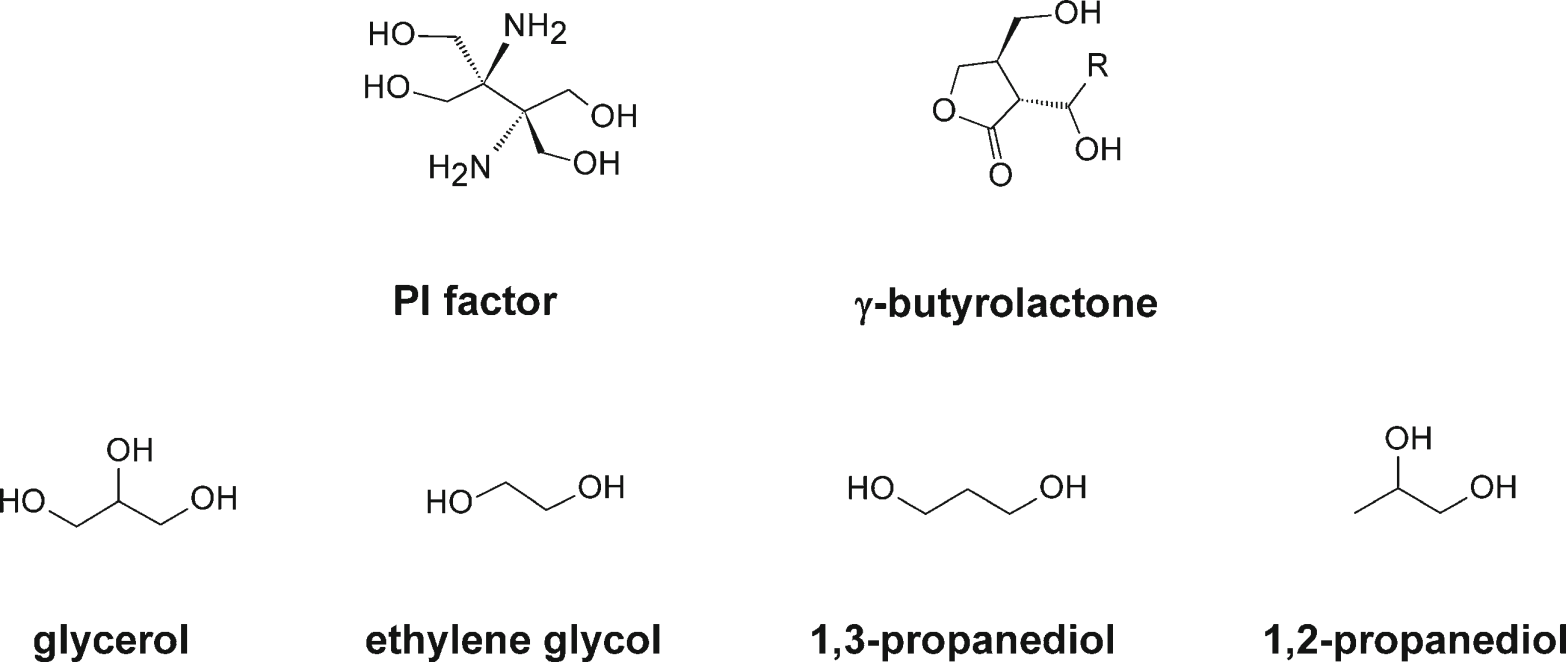
Quorum-sensing signals involved in PIM biosynthesis

*Streptomyces* use γ-butyrolactones (2,3-di-substituted-γ-butyrolactones) (GBL) as autoinducers (Fig. 6), and the signal transduction is mediated by the interaction of these autoregulators with cognate receptors which causes the receptor to dissociate from the DNA, which in turn allows transcription of the target genes. Thus, GBL receptors are transcriptional regulators belonging to the *tetR* superfamily of transcriptional factors (Gottelt et al. 2012).

GBL receptor proteins have been reported in *S. natalensis* (*sngR*) (Lee et al. 2005) and in *S. chattanoogensis* (*scgR*) (Du et al. 2011b). A GBL receptor homologue, *sprA*, has also been described in *S. chattanoogensis* (Zhou et al. 2015). Both *sngR* and *sprA*, but not *scgR*, have been described to act as positive regulators of both antifungal production and morphological differentiation (Lee et al. 2005; Zhou et al. 2015). Typically, a gene located immediately upstream and divergently from the GBL receptor encoding gene codes for the GBL synthase. Such situation has been described also in *S. natalensis* (*sngA*) (Lee et al. 2008) and *S. chattanoogensis* (*scgA*) (Du et al. 2011b). In the case of *S. chattanoogensis*, the product of a second gene immediately downstream from *scgA*, *scgX*, is also involved in GBL biosynthesis (Du et al. 2011b). The inactivation of either of the synthases *sngA* or *scgA* leads to a decrease in PIM production and a delay in morphological differentiation (Lee et al. 2008; Zhou et al. 2015) that has to be attributed to a reduction of a putative GBL in the cells. However, a true GBL was never found in *S. natalensis* (Recio et al. 2004) or *S. chattanoogensis* (Du et al. 2011b).

In *S. natalensis*, although we have not been able to detect any GBL, we discovered an inducing compound of a novel class (PI factor; 2,3-diamino-2,3-bis(hydroxymethyl)-1,4-butanediol) (Fig. 6) (Recio et al. 2004). PI factor elicits polyene production in *S. natalensis* mutants that had lost their ability to produce PIM at nanomolar concentrations in a manner characteristic of quorum sensing (Recio et al. 2004). The compound is exported out of the cells by the amino acid exporter *pimT*, and in contrast to GBLs, it is thought to be recognised at the membrane level and not intracellularly (Vicente et al. 2009). However, the exact mechanism remains unknown. Studies of PI factor have been limited by access to pure preparations of the compound. Recently, a chemical synthesis method has been reported (Morin and Sello 2010) that will permit future studies. Interestingly, *S. natalensis* seems to be able to integrate different quorum signals since A-factor from *S. griseus* (a well-known autoregulator of the GBL class) also triggers PIM production in the mutants (Recio et al. 2004).

Glycerol, ethylene glycol and 1,2 or 1,3-propanediol (Fig. 6) have also been described to elicit the production of PIM in *S. natalensis* although at higher concentrations than PI factor (Recio et al. 2006). Interestingly, glycerol also stimulated the production of seven different polyene macrolides by their respective producer strains, including *S. noursei* (nystatin), *S. rimosus* (rimocidin), *S. griseus* (candicidin), *S. filipinensis* (filipin), *S. albulus* (tetrafungin), *S. eurocidicus* (eurocidin) and *S. cinnamoneum* (fungichromin). Although the exact mechanism remains unknown, the action of glycerol seems to be independent of PI factor-inducing effect (Recio et al. 2006).

#### Cholesterol oxidase

All the gene clusters for small-sized polyenes described up to date contain a cholesterol oxidase-encoding gene. These are *pimE* and *scnE* in the case of PIM (Aparicio et al. 2000; Du et al. 2011a), *tetrO* in the case of tetramycin (Cao et al. 2012), *pteG* and *filG* in the case of filipin (Ikeda et al. 2003; Payero et al. 2015) and *rimD* in the case of rimocidin/CE-108 (Seco et al. 2004). Cholesterol oxidases (3b-hydroxysterol oxidases; EC 1.1.3.6) are flavoproteins that catalyse both the oxidation of cholesterol to 5-cholesten-3-one with the reduction of molecular oxygen to hydrogen peroxide and the isomerisation of the Δ^5^ bond to yield 4-cholesten-3-one as the final product. This enzyme participates in the initial step in the degradation of cholesterol (or other sterols with a 3-β-hydroxyl group) as a carbon and energy source for growth of different bacteria but had no obvious role in antifungal production (Aparicio and Martín 2008).

In *S. natalensis*, functional analysis studies led to the surprising finding that *pimE* is essential for the biosynthesis of PIM. This extracellular enzyme, or other cholesterol oxidases, was shown to restore PIM production when added to knockout mutant culture broths or ‘resting cells’ (Mendes et al. 2007a). Hence, it has been proposed that these enzymes could act as fungal sensors, probably via ergosterol detection and in response trigger, by an unknown mechanism, antifungal production. This would confer a selective advantage on the producing organisms, which are soil dwellers, against their fungal competitors with ergosterol-containing membranes (Aparicio and Martín 2008).

### Production improvement strategies

At industrial scale, PIM is produced by fermentation, which requires microbial strains producing high titres of the compound. Given that wild-type strains isolated from nature produce only discrete amounts of PIM, this implies the need for production improvement to meet commercial requirements. This has been traditionally achieved by sequential rounds of random mutation and selection, but the knowledge gained during the last years has provided new tools for a more rational way of yield improvement.

Different approaches have been used to increase PIM production yield. These studies include optimisation of medium composition (Farid et al. 2000; Chen et al. 2008), improvement of cultivation conditions (El-Enshasy et al. 2000; Liang et al. 2008) and strain gene manipulation. One of the most straightforward ways to improve metabolite production by gene manipulation is the overexpression of poorly expressed activators, and that turned out to be the case of *pimM* in *S. natalensis*. Gene dosage increment of *pimM* by using integrative vectors resulted in increased PIM production, thus suggesting that its expression constitutes a bottleneck for antifungal production (Antón et al. 2007). Moreover, PAS-LuxR regulators are functionally equivalent, to the extent that introduction of a single copy of *pimM* (or other PAS-LuxR regulator) into the chromosome of a given polyene producer boosts the production of the corresponding polyene (Santos-Aberturas et al. 2011b). These findings constituted the first report of a general mechanism regulating polyene production, and established the rationale for enhancement of PIM, and other polyene production in different producers of these antifungals (Du et al. 2009; Wei et al. 2011; Wu et al. 2014). Improvement in production varied between strains and the production medium used but ranged from 40% in the rimocidin producer *S. rimosus* (Santos-Aberturas et al. 2011b) to 460% in the PIM producer *S. chattanoogensis* (Du et al. 2009) (Table 2). These strategies for production improvement have used the native promoter of the PAS-LuxR gene, but the use of constitutive promoters such as ermE*p instead of the original promoter, and in addition to the native promoter (double promoter), have also been described with improved results (Wei et al. 2011; Wu et al. 2014).

Other strategies reported for production improvement have included the overexpression of phosphopantetheinyl transferases (Jiang et al. 2013a, b), the GBL synthase *sngA* (Lee et al. 2008) and pleiotropic regulators such as *whiG* (Liu et al. 2015a) or *wblA* (Yu et al. 2014) or deletion of the two-component system *phoRP* (Mendes et al. 2007b) and the GBL receptor *sngR* (Lee et al. 2005) (Table 2).

The addition of short-chain carboxylic acids has also been reported to increase PIM yield and also to shorten production time. Acetic and propionic acids were particularly stimulatory in *S. natalensis* cultures, especially when added together in a 7:1 ratio (total concentration 2 g/L) (Elsayed et al. 2013). This makes sense since these compounds are precursors of polyketide biosynthesis. Similarly, propanol has also been reported as stimulatory (Li et al. 2014) (Table 2).

Other successful strategies used included genome shuffling by recursive protoplast fusion, reaching increments of 97% in comparison with the parental strain (Luo et al. 2012), the chromosomal integration of the *Vitreoscilla* hemoglobin *vgb* gene, which rendered a maximum of 407 % increase depending on the growth conditions (Wang et al. 2014b), or the use of fungal elicitors present in extracellular extracts of *A. niger* and in particular of *P. chrysogenum* (Wang et al. 2013) (Table 2).

### Prospects

Amongst the major applications of PIM, its use as a natural food antimicrobial is generalised worldwide, being used in more than 150 countries. PIM has been used for more than 40 years now and continues to constitute a gold standard in the preservation of foods and beverages against mould spoilage and the inherent risk of mycotoxin poisoning. Its broad spectrum of activity against almost every type of fungi but not bacteria, its lack of effect on the quality of food, its low likelihood of causing microbial resistance and especially its safeness for consumption have made this molecule an ideal biopreservative. Thus, its use that was initially restricted to the surface treatment of some types of cheese has expanded exponentially to other foods, beverages, storage facilities and even some crops. Furthermore, the increasing demand of healthy processed foods and beverages with natural antimicrobials as alternatives to physical- and chemical-based antimicrobial treatments ensures that PIM use will continue growing in the future.

Thanks to the advances in the genetic manipulation of PIM producers and the considerable progress in the knowledge about the regulation of PIM production; several PIM derivatives with improved properties have been developed, and many of the bottlenecks hampering PIM production at high titres have been overcome. The future application of this knowledge at the industrial scale will hopefully permit to satisfy the growing PIM commercial demands. In this sense, the present availability of the genome sequences of PIM producers such as *S. natalensis* (GenBank JRKI01; Beites et al. 2015) and *S. chattanoogensis* (GenBank LGKG01) will certainly constitute a very valuable tool in the efforts to improve PIM production.
